# Epidemiology and Related Risk Factors of Preterm Labor as an obstetrics emergency

**Published:** 2017-01-08

**Authors:** Ali asghar Halimi asl, Saeed Safari, Mohsen Parvareshi Hamrah

**Affiliations:** 1 Department of Pediatrics, Shohadaye Tajrish Hospital, Shahid Beheshti University of Medical Sciences, Tehran, Iran.; 2 Department of Emergency, Shohadaye Tajrish Hospital, Shahid Beheshti University of Medical Sciences, Tehran, Iran.

**Keywords:** Premature birth, infant, premature, obstetric labor, premature, fetal membranes, premature rupture, emergencies

## Abstract

**Introduction:**

Preterm birth is still a major health problem throughout the world, which results in 75% of neonatal mortality. Preterm labor not only inflicts financial and emotional distress, it may also lead to permanent disability. The present study was conducted to determine the related risk factors and preventive measures of preterm labor.

**Methods:**

This retrospective cross-sectional study assessed all preterm labors, as well as an equal number of term labors, during seven years, at an educational hospital. Probable risk factors of preterm labor were collected using medical profiles of participants by the aid of a pre-designed checklist. Significant related factors of preterm labor were used for multivariate logistic regression analysis with SPSS 21.0.

**Result:**

810 cases with the mean age of 28.33 ± 6.1 years were evaluated (48.7% preterm). Multipartite; fetal anomaly; prenatal care; smoking; not consuming folic acid and iron supplements; in vitro fertilization; history of infertility, caesarian section, trauma, systemic disease, and hypertension; amniotic fluid leak; rupture of membranes; cephalic presentation; vaginal bleeding; placenta decolman; oligohydramnios; pre-eclampsia; chorioamnionitis; uterine abnormalities; cervical insufficiency; intercourse during the previous week; short time since last delivery; and mother’s weight significantly correlated with preterm labor.

**Conclusion:**

Based on the results of the present study, intercourse during the previous week, multipartite, short time from last delivery, preeclampsia, fetal anomaly, rupture of membranes, hypertension, and amniotic fluid leak, respectively, were risk factors for preterm labor. On the other hand, iron consumption, cephalic presentation, systematic disease, history of caesarian section, prenatal care, and mother’s weight could be considered as protective factors.

## Introduction

Preterm labor is an obstetrics emergency and a threat to population health. 75% of infant mortality is related to preterm labor (1, 2). Preterm labor not only inflicts financial and emotional distress on the family, it may also lead to permanent disability (physical or neural damages) in infants.

Approximately one-third of preterm labor survivors suffer from severe long–term neurological disabilities, such as cerebral palsy or mental retardation (3). Furthermore, preterm infants carry increased risk of a range of neurodevelopmental impairments and disabilities including behavioral problems, school learning difficulties, chronic lung disease, retinopathy of prematurity, hearing impairment, and lower growth attainment (4). 

Over the last two decades, preterm birth rate has remained unchanged or even risen in most countries, despite the increased understanding of possible risk factors and their pathological mechanisms (5-7). 

Although neonatal mortality rate has fallen globally between 1990 and 2009 (8), the absolute number and rate of preterm births has increased during this period. Preterm birth was the second leading cause of death in children under 5 years old (9). In 2013, preterm birth rate in Germany, Brazil and United States were 8.7%, 10.7 and 12%, respectively (10, 11). The vast majority (85%) of global preterm births occur in Asia and Africa, where health systems are weak and inadequate (12, 13). In Iran incidence of preterm labor was 7.2% in Tehran, 5.5% in Shiraz, and 8.4% in Khorramabad (14-16). 

Although in most cases preterm births occur idiopathically, fetal, uterine, and placental factors as well as maternal chronic diseases, can affect preterm birth (17). In the USA, 70% of preterm births were idiopathic and the rest were due to pre-eclampsia (50%), fetal distress (25%) and abruption (25%) (18). In another study, preterm multifetal pregnancies and hypertension were introduced as the major factors affecting preterm birth (19). In order to determine the incidence and etiologic factors of preterm labor, the present study was conducted on newborns at the obstetrics emergency department of Shohadaye Tajrish Hospital with a view to identifying preventive measures.

## Methods

***Study design and setting***

This retrospective cross-sectional study assessed all preterm labors during seven years, from March 2008 until March 2015, at Shohadaye Tajrish Hospital, Tehran, Iran, by normal vaginal delivery or cesarean section, using census method. An equal number of term labors were selected by simple random sampling as the control group. The study protocol was approved by the Ethical Committee of Shahid Beheshti University of Medical Sciences. The researchers adhered to the principles of Helsinki Declaration, as well as confidentiality of patient data and patient rights.

***Data gathering***

Probable risk factors of preterm labor such as: mother’s age, weight, body mass index, and job; type of delivery (natural or caesarian section), baby’s sex and weight; apgar score at 1 and 5 minutes; multi-partite; fetal abnormalities; prenatal care; smoking, alcohol, and opium abuse; history of folic acid, metformin, and iron consumption; history of in vitro fertilization, infertility, abortion, preterm delivery, trauma, vaginal bleeding, intra uterine fetal death (IUFD), dental infection, respiratory infection, and caesarian section; amniotic fluid leak; rupture of membranes; cephalic presentation; vaginal infection; placenta decolman; placenta praevia; polyhydramnios; oligohydramnios; urinary tract infection; systemic disease; anemia; hypertension; preeclampsia; eclampsia; chorioamnionitis; uterine abnormalities, cervical insufficiency; placental insufficiency; polycystic ovary; history of intercourse during the previous week; and time from last delivery were collected using medical profiles of participants by the aid of a pre-designed checklist. Incomplete patient files were excluded. Short time from last delivery was considered to be 1 year.

**Statistical analysis**

The data were analyzed with SPSS software version 21.0. Qualitative data were reported as mean ± standard deviation and quantitative ones as frequency and percentage. Frequency of all risk factors were compared between the two groups (preterm and term) using chi square and Fisher’s exact tests. Multivariate logistic regression analysis was applied to independent statistically significant factors for developing a predictive model and odds ratio (OR) of each risk factor was calculated. P value under 0.05 was considered significant.

## Results

810 cases with the mean age of 28.33 ± 6.1 (14 - 64) years were evaluated (48.7% preterm). Table 1 depicts baseline characteristics of the studied patients. Among the studied risk factors, multipartite (p < 0.001), fetal anomaly (p = 0.022), prenatal care (p = 0.005), smoking (p = 0.004), not consuming folic acid (p = 0.004), not consuming iron supplements (p < 0.001), in vitro fertilization (p = 0.014), history of infertility (p = 0.005), amniotic fluid leak (p < 0.001), rupture of membranes (p < 0.001), history of caesarian section (p < 0.001), cephalic presentation (p < 0.001), history of trauma (p = 0.015), vaginal bleeding (p < 0.001), placenta decolman (p = 0.003), oligohydramnios (p < 0.001), history of systemic disease (p < 0.001), history of hypertension (p = 0.006), pre-eclampsia (p = 0.001), chorioamnionitis (p = 0.003), uterine abnormalities (p = 0.034), cervical insufficiency (p = 0.001), intercourse during the previous week (p < 0.001), short time since last delivery (p = 0.040), and mother’s weight (p = 0.012) significantly correlated with higher risk of preterm labor. Table 3 shows the results of multivariate logistic regression analysis. Intercourse during the previous week (OR: 23.1), multipartite (OR: 21.8), short time from last delivery (OR: 4.8), pre-eclampsia (OR: 4.7 ), fetal anomaly (OR: 3.6), rupture of membranes (OR: 3.5), hypertension (OR: 3.3), and amniotic fluid leak (OR: 2.1), respectively, were risk factors and iron consumption (OR: 0.3), cephalic presentation (OR: 0.4), systematic disease (OR: 0.6), history of caesarian section (OR: 0.6), prenatal care (OR: 0.6), and mother’s weight (OR: 0.98), respectively, were preventive factors of preterm labor.

## Discussion

Based on the findings of the present study, independent related factors of preterm labor were multipartite, fetal anomaly, prenatal care, smoking, not consuming folic acid, not consuming iron supplements, in vitro fertilization, history of infertility, amniotic fluid leak, rupture of membranes, history of caesarian section, cephalic presentation, history of trauma, vaginal bleeding, placenta decolman, oligohydramnios, history of systemic disease, history of hypertension, preeclampsia, chorioamnionitis, uterine abnormalities, cervical insufficiency, intercourse during the previous week, short time since last delivery, and mother’s weight. Intercourse during the previous week, multipartite, short time from last delivery, preeclampsia, fetal anomaly, rupture of membranes, hypertension, and amniotic fluid leak, respectively, were risk factors for preterm labor. On the other hand, iron consumption, cephalic presentation, systematic disease, history of caesarian section, prenatal care, and mother’s weight could be considered as protective factors.

Preterm labor, as mentioned before, is a major obstetric and pediatric challenge because it is a common, persistent, and often devastating condition with considerable medical, economic, emotional, and social impact (20). It is thought to be a syndrome initiated by multiple mechanisms, consisting of infection or inflammation, uteroplacental ischaemia or haemorrhage, uterine overdistension, stress, and other immunologically mediated processes. However, a defined mechanism cannot be established in most cases (21). Despite advances in understanding risk factors and mechanisms related to preterm labor, the preterm labor rate has risen in most industrialized countries. In the USA, preterm labor rate increased from 9.5% in 1981 to 12.7% in 2005 (22, 23). In the present study, low maternal weight has increased the risk of preterm labor, while in retrospective studies, this factor weakly correlated with preterm birth (24-26). Although most of the term births were via natural delivery and most of the preterm labors via caesarian delivery, no significant relationship was found. The mean age of mothers with preterm labor in this study, were the same as mothers with term infants, while the incidence of prematurity in different studies was greater in old mothers (27, 28). Several studies have demonstrated that adequate utilization of pre-natal care is accompanied with improved birth weights and lower risk of preterm birth. On the other hand, inadequate pre-natal care is often referred to as a risk factor for poor pregnancy outcomes. In our study, women who had no well-designed pre-natal care program, were at risk for preterm labor (29, 30). Infections and vaginosis are well-known risk factors for preterm birth. In a study, presence of bacterial vaginosis at 28 weeks gestation was associated with an increased risk of spontaneous preterm birth (31). Nevertheless, these factors were not associated with preterm birth in our study. Antibiotic therapy could either eliminate infections or modify their effects on pregnancy outcome (32-34). Smoking has been linked to preterm labor, and in this study this factor had an association with it (35, 36). Although sexual activity, particularly intercourse, during pregnancy has been connected to preterm labor, because of direct effects of semen on initiating preterm labor or alteration of vaginal pH, there is evidence that shows sexual activity during pregnancy is not associated with preterm birth. In this study, intercourse during the previous week affected preterm birth (37). High levels of alcohol consumption during pregnancy have obvious adverse effects on fetal development, but in this project there is no consistency between use of alcohol and chance of preterm birth (38). Various studies have suggested lower rates of preterm birth in women taking dietary supplements (39). Dietary supplements taken before, but not after conception, were linked with a reduced rate of preterm birth; however, a placebo-controlled trial of vitamin supplements in women before conception and 2 months after pregnancy, reported no effect on preterm birth rate (40, 41). Our results showed that folic acid and iron consumption significantly decrease the rate of preterm birth. Preterm rupture of fetal membranes leads to 30% of preterm births in industrialized countries. Management, consists of maternal and fetal surveillance for labor, infection, and abruption, and administration of corticosteroids or antibiotics (42, 43). Ruptures of the fetal membranes are remarkably seen in preterm birth. The availability of medical reproductive techniques has increased the number of multiple pregnancies. In addition, multiple pregnancies resulting from reproductive medical treatments are more common in women of advanced maternal age (44). The preterm birth rate for multiple pregnancies stands at 40 - 60% (45). Multipartite and in vitro fertilization directly correlated with preterm birth. In our study, pre-eclampsia was 72.3% in preterm labor and 27.7% in term labors. In our study, history of chronic hypertension was seen in 59.4% of mothers with preterm labor and 40.6% in mothers with term labor. In other studies the most common maternal disease was hypertension (16).

**Table 1:** Baseline characteristics of studied patients based on age of delivery

**Variable**	**Term**	**Preterm**	**P value**
**Age (year)**	28.25 ± 5.9	28.37 ± 6.34	0.766
**Weight (Kg)**	76.38 ± 13.11	73.78 ± 14.19	0.012
**Job**			
Home keeper	319 (46.9)	361 (53.1)	0.626
Employee	7 (50)	7 (50)	
**Type of delivery**			
Natural	302 (54)	257 (46)	0.002
Caesarian section	101 (42.8)	135 (57.2)	
**Baby’s Sex**			
Boy	205 (46.8)	233 (53.2)	0.065
Girl	195 (55.1)	159 (44.9)	
**Baby’s weight (gram)**	3184 ± 542	2080 ± 1012	< 0.001
**Apgar (1** ^st^ ** minute)**	7.1 ± 2.3	5.7 ± 3.0	0.076
**Apgar (5** ^th^ ** minute)**	8.9 ± 0.6	7.9 ± 2.4	< 0.001

Data are presented as mean ± standard deviation or number (%).

**Table 2:** Comparison of studied risk factors of preterm delivery between term and pre term pregnancy

**Risk factor**	**Term n (%)**	**Preterm n (%)**	**P value**
**Multipartite**			
Yes	3 (6.7)	42 (93.3)	< 0.001
No	403 (53)	357 (47)	
**Fetal anomaly**			
Yes	8 (29.6)	19 (70.4)	0.022
No	398 (51.2)	380 (48.8)	
**Prenatal care**			
Yes	159 (56.8)	121 (43.2)	0.005
No	247 (47)	278 (53)	
**Smoking**			
Yes	0 (0)	8 (100)	0.004
No	406 (50.9)	391 (49.1)	
**Alcohol usage**			
Yes	0 (0)	1 (100)	0.496
No	406 (50.5)	398 (49.9)	
**Opium usage**			
Yes	4 (28.6)	10 (71.4)	0.083
No	402 (50.8)	389 (49.2)	
**Folic acid consumption**			
Yes	149 (57.3)	111 (42.7)	0.004
No	257 (47.2)	288 (52.8)	
**Metformin consumption**			
Yes	5 (62.5)	3 (37.5)	0.372
No	401 (50.3)	396 (49.7)	
**Iron consumption**			
Yes	371 (55.1)	302 (44.9)	< 0.001
No	35 (26.5)	97 (73.5)	
**In vitro fertilization**			
Yes	6 (26.1)	17 (73.9)	0.014
No	400 (51.2)	382 (48.8)	
**History of infertility**			
Yes	26 (35.6)	47 (64.4)	0.005
No	380 (51.9)	352 (48.1)	
**History of abortion**			
Yes	71 (51.1)	68 (48.9)	0.471
No	335 (20.3)	331 (49.7)	
**History of preterm delivery**			
Yes	8 (36.4)	14 (63.6)	0.131
No	398 (50.8)	385 (49.2)	
**History of IUFD**			
Yes	8 (36.4)	14 (63.6)	0.131
No	398 (50.8)	385 (49.2)	
**Amniotic fluid leak**			
Yes	79 (33.3)	158 (66.7)	<0.001
No	327 (57.6)	241 (42.4)	
**Rupture of membranes **			
Yes	30 (22.9)	101 (77.1)	< 0.001
No	376 (55.8)	298 (44.2)	
**History of caesarian section**			
Yes	142 (65.1)	76 (34.9)	< 0.001
No	264 (45)	323 (55)	
**Cephalic presentation**			
Yes	343 (56.6)	263 (43.3)	< 0.001
No	63 (31.7)	136 (68.3)	
**History of trauma**			
Yes	3 (20.3)	12 (80)	0.015
No	403 (51)	387 (49)	
**History of surgery**			
Yes	34 (54)	29 (46)	0.326
No	372 (50.1)	370 (49.9)	
**Vaginal bleeding**			
Yes	6 (18.8)	26 (81.3)	< 0.001
No	400 (51.7)	373 (48.3)	
**Vaginal infection**			
Yes	5 (35.7)	9 (64.3)	0.200
No	401 (50.7)	390 (49.3)	
**Placenta decolman**			
Yes	3 (16.7)	15 (83.3)	0.003
No	403 (51.2)	384 (48.8)	
**Placenta praevia**			
Yes	4 (40)	6 (60)	0.365
No	402 (50.6)	393 (49.4)	
**Polyhydramnios**			
Yes	3 (37.5)	5 (62.5)	0.353
No	403 (50.6)	394 (49.4)	
**Oligohydramnios**			
Yes	12 (25)	36 (75)	< 0.001
No	394 (52)	363 (48)	
**Urinary tract infection**			
Yes	65 (56.5)	50 (43.5)	0.095
No	341 (49.4)	349 (50.6)	
**Systemic disease**			
Yes	133 (60.2)	88 (39.8)	< 0.001
No	273 (46.7)	311 (53.3)	
**Anemia**			
Yes	31 (51.7)	29 (48.3)	0.475
No	375 (50.3)	370 (49.7)	
**History of hypertension**			
Yes	58 (40.6)	85 (59.4)	0.006
No	348 (52.6)	314 (47.4)	
**Preeclampsia**			
Yes	13 (27.7)	34 (72.3)	0.001
No	393 (51.8)	385 (48.2)	
**Eclampsia**			
Yes	1 (20)	4 (80)	0.181
No	405 (50.6)	395 (49.4)	
**Chorioamnionitis**			
Yes	1 (8.3)	11 (91.7)	0.003
No	405 (51.1)	388 (48.9)	
**Uterine abnormalities**			
Yes	6 (28.6)	15 (71.4)	0.034
No	400 (51)	384 (49)	
**Cervical insufficiency**			
Yes	0 (0)	10 (100)	0.001
No	406 (51.1)	389 (48.9)	
**Placental insufficiency**			
Yes	0 90)	3 (10)	0.121
No	406 (50.6)	396 (49.4)	
**Polycystic ovary**			
Yes	1 (33.3)	2 (66.7)	0.493
No	405 (50.5)	397 (49.5)	
**Body mass index**			
Yes	12 (63.2)	7 (36.8)	0.187
No	394 (50.1)	392 (49.9)	
**Intercourse during the previous week**			
Yes	1 (6.3)	15 (93.8)	< 0.001
No	405 (51.3)	384 (48.7)	
**Short time since last delivery **			
Yes	7 (30.4)	16 (69.6)	0.040
No	399 (51)	383 (49)	
**History of dental infection**			
Yes	1 (14.3)	6 (85.7)	0.59
No	405 (50.8)	393 (49.2)	
**History of respiratory infection**			
Yes	2 (50)	2 (500	0.681
No	404 (50.4)	397 (49.6)	

IUFD: Intrauterine fetal death.

**Table 3:** The results of multivariate logistic regression analysis

**Variable**	**Odds ratio (95% CI** * **)**	**P value**
**Intercourse during the previous week**	23.1 (2.7-194.2)	0.004
**Multipartite**	21.8 (4.8-97.9)	<0.001
**Short time from last delivery **	4.8 (1.4-16.2)	0.012
**Preeclampsia**	4.7 (1.9-11.6)	0.001
**Fetal anomaly**	3.6 (1.1-11.2)	0.024
**Rupture of membranes **	3.5 (2-6.2)	<0.001
**Hypertension**	3.3 (1.9-5.5)	<0.001
**Amniotic fluid leak**	2.1 (1.4-3.4)	0.001
**Mother’s Weight**	0.98 (0.96-0.99)	0.005
**Prenatal care**	0.6 (0.04-0.09)	0.036
**History of caesarian section**	0.6 (0.4-0.9)	0.020
**Systematic Disease**	0.6 (0.4-0.9)	0.010
**Cephalic presentation**	0.4 (0.2-0.6)	<0.001
**Iron consumption**	0.3 (0.2-0.6)	<0.001

* CI: confidence interval.

Using the results of this study and similar ones to eliminate the risk factors and reinforce the protective factors would be helpful in decreasing the rate of preterm labor and its human and social burden. Yet, for accurately determining these factors, studies with better design, such as cohort studies, with proper follow-up period and large study population, are needed. Since the studied hospital is a referral center for these patients, it represents the general population of the country to a great extent. Still, the final decision regarding factors definitely affecting pre-term labor should be made after further studies.

## Conclusion:

Based on the results of the present study, intercourse during the previous week, multipartite, short time from last delivery, preeclampsia, fetal anomaly, rupture of membranes, hypertension, and amniotic fluid leak, respectively, were risk factors for preterm labor. On the other hand, iron consumption, cephalic presentation, systematic disease, history of caesarian section, prenatal care, and mother’s weight could be considered as protective factors.
